# Monoamine Oxidase-A Occupancy by Moclobemide and Phenelzine: Implications for the Development of Monoamine Oxidase Inhibitors

**DOI:** 10.1093/ijnp/pyv078

**Published:** 2015-08-27

**Authors:** Lina Chiuccariello, Robert G Cooke, Laura Miler, Robert D Levitan, Glen B Baker, Stephen J Kish, Nathan J Kolla, Pablo M Rusjan, Sylvain Houle, Alan A Wilson, Jeffrey H Meyer

**Affiliations:** CAMH Research Imaging Centre and Campbell Family Mental Health Research Institute, Centre for Addiction and Mental Health and Departments of Psychiatry, Pharmacology and Toxicology, and Institute of Medical Sciences, University of Toronto, Canada (Drs Chiuccariello, Cooke, Levitan, Kish, Kolla, Rusjan, Houle, Wilson, and Meyer, and Ms Miler); Department of Psychiatry (NRU) and Neuroscience and Mental Health Institute, University of Alberta, Edmonton, Canada (Dr Baker).

**Keywords:** Moclobemide, monoamine oxidase-A, monoamine oxidase inhibitors, phenelzine, positron emission tomography

## Abstract

**Background:**

Monoamine oxidase inhibitors (MAOIs) are being developed for major depressive disorder, Alzheimer’s, and Parkinson’s Disease. Newer MAOIs have minimal sensitivity to tyramine, but a key limitation for optimizing their development is that standards for *in vivo* monoamine oxidase-A (MAO-A) occupancy in humans are not well established. The objectives were to determine the dose-occupancy relationship of moclobemide and the occupancy of phenelzine at typical clinical dosing.

**Methods:**

Major depressive episode (MDE) subjects underwent [^11^C]harmine positron emission tomography scanning prior to and following 6 weeks of treatment with moclobemide or phenelzine.

**Results:**

Mean brain MAO-A occupancies were 74.23±8.32% for moclobemide at 300–600mg daily (n = 11), 83.75±5.52% for moclobemide at 900–1200mg daily (n = 9), and 86.82±6.89% for phenelzine at 45–60mg daily (n = 4). The regional dose-occupancy relationship of moclobemide fit a hyperbolic function [F(x) = a(x/[b + x]); F_(1,18)_ = 5.57 to 13.32, *p* = 0.002 to 0.03, mean ‘a’: 88.62±2.38%, mean ‘b’: 69.88±4.36 mg]. Multivariate analyses of variance showed significantly greater occupancy of phenelzine (45–60mg) and higher-dose moclobemide (900–1200mg) compared to lower-dose moclobemide [300–600mg; F_(7,16)_ = 3.94, *p* = 0.01].

**Conclusions:**

These findings suggest that for first-line MDE treatment, daily moclobemide doses of 300–600mg correspond to a MAO-A occupancy of 74%, whereas for treatment-resistant MDE, either phenelzine or higher doses of moclobemide correspond to a MAO-A occupancy of at least 84%. Therefore, novel MAO inhibitor development should aim for similar thresholds. The findings provide a rationale in treatment algorithm design to raise moclobemide doses to inhibit more MAO-A sites, but suggest switching from high-dose moclobemide to phenelzine is best justified by binding to additional targets.

## Introduction

Monoamine oxidase inhibitors (MAOIs) are best known for their antidepressant effects, but their recently elucidated role in influencing oxidative stress and apoptosis (Ou et al., 2006a; Fitzgerald et al., 2007; Johnson et al., 2011) has led to investigations of MAOIs for the treatment of neurodegenerative diseases, such as Alzheimer’s and Parkinson’s Disease (Maruyama et al., 2003; Youdim et al., 2006). This resurgence in MAOI development has led to compounds with improved tolerability profiles compared to the initial irreversible MAOIs, particularly in regard to tyramine-induced hypertension and dietary restrictions (Yanez et al., 2012). This improved tolerability is being achieved through several strategies, which include greater selectivity for either monoamine oxidase-A (MAO-A) or monoamine oxidase-B (MAO-B), reversible binding to MAO, and/or use of a pro-drug design in which the initial drug is metabolized to the active drug in the brain, resulting in an increased ratio of brain-to-gut concentration of the active drug (Maruyama et al., 2003; Gal et al., 2005; Avramovich-Tirosh et al., 2007).

Development of new MAOIs could be accelerated if the meaningful threshold measures of occupancy data were established. Presently, target engagement of novel MAOIs is largely based on *in vitro* binding characteristics and inhibition of MAO in other species, rather than *in vivo* occupancy measurement in humans (Bergstrom et al., 1997b; Bottlaender et al., 2010). While these are important assessments, occupancy measurement can also provide valuable information for *in vivo* translation. For example, despite a 100-fold variation of *in vitro* affinity across five common selective serotonin reuptake inhibitor (SSRI) treatments, it was demonstrated using [^11^C] carbon 11-labeled 3-amino-4-(2-dimehtylaminomethyl-phenylsulfanyl)-benzonitrile positron emission tomography (PET) that serotonin transporter occupancy was approximately 80% for all medications at doses that significantly differed from placebos in double-blind randomized clinical trials (Meyer et al., 2001, 2004; Suhara et al., 2003). Inhibition of MAO in other species is important to verify that there is a reasonable brain penetration, but it cannot be assumed that this is equivalent between humans and other species, since the active efflux transporters that remove drugs from the central nervous system may differ. For the MAO-A target, [^11^C]harmine PET may be applied for occupancy measurement since [^11^C]harmine has outstanding properties as a radioligand: [^11^C]harmine is a MAO-A selective, reversible radiotracer with a high affinity for the MAO-A enzyme (Bergstrom et al., 1997a) that binds to the center of the functionally active pocket of MAO-A (Son et al., 2008). It also has high brain uptake in humans and has polar metabolites that do not cross the blood-brain barrier (for further review of [^11^C]harmine properties see Meyer et al., 2006, 2009). The affinity of harmine is approximately 1–5nM at MAO-A (Bergstrom et al., 1997b; Schwarz et al., 2003) and greater than 1000nM at MAO-B (Schwarz et al., 2003). Blocking studies with selective MAO-A inhibitors suggest complete displacement of the specific binding of [^11^C]harmine in baboons (Bergstrom et al., 1997a). However, more information is needed to determine the optimal benchmark occupancy thresholds of MAOIs to guide antidepressant development.

The main aims of this study were to determine the dose-occupancy relationship of moclobemide and to determine the occupancy of phenelzine at typical clinical dosing by using [^11^C]harmine PET before and after a 6 week trial. Moclobemide is selective for and reversibly binds to MAO-A, whereas phenelzine irreversibly binds to MAO-A and MAO-B, increases brain gama-aminobutyric acid (GABA) levels, and inhibits primary amine oxidase (semicarbazide-sensitive amine oxidase; Baker et al., 1991; Holt et al., 2004). To date, there has been limited characterization of MAO-A occupancy for antidepressants: moclobemide dosing at a total daily dose of 600mg has an average occupancy of 75% after either 1 week or 6 weeks of treatment (Ginovart et al., 2006; Sacher et al., 2011). CX157 (TriRima), a novel selective MAO-A inhibitor in development, had an occupancy of 47 to 72% at its phase 2 stage of development (Fowler et al., 2010). Moclobemide, given at 300 to 600mg as a total daily dose, is approved as a first-line treatment in some guidelines (Lam et al., 2009) and is considered equivalently effective and tolerable to other first-line antidepressants in other guidelines (Ellis, 2004; National Collaborating Centre for Mental Health, 2010; Baghai et al., 2011), whereas phenelzine or high-dose moclobemide is considered an option for major depressive episodes (MDE) after at least several treatments of different classes (American Psychiatric Association, 2010). Our first hypothesis was that moclobemide occupancy would be increased with a greater total daily dose. Our second hypothesis was that there would be distinct target occupancies corresponding to their clinical use, such that the higher doses of moclobemide (900mg to 1200mg total daily dose) and phenelzine treatment (45mg to 60mg total daily dose) used in treatment-resistant MDE would be associated with greater MAO-A occupancy than the lower daily dose of moclobemide (300 to 600mg total daily dose).

## Methods

### Participants and Treatment

Each participant underwent two PET scans: a baseline PET scan prior to treatment and a second scan after 6 weeks at the assigned treating dose of a MAOI. Participants who were currently taking a SSRI were first tapered off of the medication and remained medication-free for 2 weeks prior to the first PET scan and treatment with an MAOI. Participants were treated by psychiatrists at the Centre for Addiction and Mental Health (Drs Meyer, Cooke, and Levitan). Twenty-five MAOI treatment trials were completed. Subjects received an open trial of moclobemide at a dose of 150mg twice per day (bid), 300mg bid, 450mg bid, or 600mg bid, or phenelzine at a dose of 45 to 60mg assigned in bid dosing (15mg each morning (qAM) and 30mg each evening (qhs) or 30mg bid). For assigned treating doses of moclobemide greater than 150mg bid, titrating was done by raising the dose by 150mg bid each week. For phenelzine treatment, initial dosing was 15mg once per day for week 1, 15mg bid for week 2, and 15mg qAM with 30mg qhs for week 3. Subjects who seemed unlikely to tolerate a higher dose stayed at this dose of phenelzine whereas subjects deemed likely to tolerate 30mg bid were subsequently raised to this dose.

To obtain data in a manner consistent with usual clinical practice, assignment to treatment was based upon clinical history: for those with no previous antidepressant treatment or previous non-response to at least one antidepressant treatment, a total daily dose of 300 to 600mg of moclobemide was administered. For those with a history of non-response to at least two antidepressant trials or a history of non-response to low dose moclobemide, a total daily dose of 900 to 1200mg of moclobemide was assigned. Those with histories of non-response to three antidepressant trials of at least two different classes or non-response to two antidepressant classes if one trial included moclobemide were offered a clinical trial of phenelzine (45–60mg). Two subjects were permitted to take a lower dose since they did not tolerate the dose assigned (one subject took 150mg qAM and 300mg qHS rather than 300mg bid of moclobemide and one subject took phenelzine at 7.5mg qAM and 15mg qHS). The participant that was lowered to a total daily dose of 22.5mg of phenelzine was excluded from the analyses (and not included in the analyses presented in the abstract) because the aim of accumulating phenelzine MAO-A occupancy data was to characterize the occupancy at typical treating doses. For the 25 treatment trials, 21 participants with MDE were recruited (mean age = 35.6, standard deviation = 9.3). Two subjects who did not respond to 300mg and 900mg moclobemide opted to subsequently re-enroll and obtain treatment with 600mg and 1200mg moclobemide, respectively. Furthermore, two participants who did not respond to either a high dose (1200mg) of moclobemide or phenelzine opted to re-enroll for subsequent treatment with the alternative medication. The participant that was lowered to a total daily dose of 22.5mg of phenelzine was excluded from the analyses, hence there were 25 subjects but 24 treatment trials included in the analyses. Participants had follow-up visits approximately every 2 weeks with the treating psychiatrist (Drs Meyer, Cooke, or Levitan) and research associate (Dr Chiuccariello) while taking medication to ensure tolerance and compliance (the latter verified by metabolites in urine drug screen and plasma sampling). Compliance was additionally verified through a plasma sample, which was taken on the second scan day (phenelzine: 1.15±1.19ng/ml, moclobemide: 3795.4±2336.5ng/ml). A detailed description of the assay used to quantify phenelzine and moclobemide levels may be found in the supplemental section. Participant demographics, clinical histories, and treatment responses are shown in Table 1.

**Table 1.** Demographics, Clinical History, and Treatment Response for Treatment Groups

	Moclobemide Group		Phenelzine Group	Significance
	Moclobemide Dose (300–600mg, n = 11)	Moclobemide Dose (900– 1200mg, n = 9)	Phenelzine Dose (45–60mg, n = 5)	
Demographics				
Age, mean (SD)	32.6 (8.3)	36.4 (9.8)	42.8 (6.2)	F _(2,24)_ = 2.5, *p* = 0.1
No. Male,Female	2,9	5,4	3,2	X^2^_(2)_ = 3.9, *p* = 0.1
Clinical History				
Age of onset, mean (SD)	20.4 (9.8)	23.6 (10.3)	27.8 (10.8)	F_(2,24)_ = 0.9, *p* = 0.4
No. of previous episodes, mean (SD)	3.6 (4.9)	1.9 (1.3)	1.2 (0.5)	F_(2,24)_ = 1.0, *p* = 0.4
No. with reversed neurovegetative symptoms	2	2	1	X^2^_(2)_ = 0.05, *p* = 1.0
No. of previous antidepressant trials, mean (SD)	1.7 (1.9)	3.7 (2.1)	5 (4.3)	F_(2,24)_ = 3.1, *p* = 0.06
No. of antidepressant classes in previous trials, mean (SD)	1.2 (1.3)	2.6 (1.1)	3.0 (2.1)	F_(2,24)_ = 3.6, *p* = 0.04
Treatment Response				
HRSD score pre-treatment, mean (SD)	20.7 (5.3)	21.2 (3.1)	21.2 (3.9)	F_(2,24)_ = 0.04, *p* = 1.0
HRSD score post-treatment, mean (SD)	12.0 (4.0)	14.3 (8.5)	9.0 (6.9)	F_(2,24)_ = 1.1, *p* = 0.4

HRSD, Hamilton Rating Scale for Depression; SD, standard deviation.

All participants met criteria for a current MDE and major depressive disorder (MDD) as determined by the Structured Clinical Interview for DSM-IV Axis I disorders (First et al., 1996) by an experienced rater and verified by subsequent consultation with a psychiatrist (Drs Meyer, Cooke, or Levitan). General inclusion criteria included good physical health and a Hamilton Rating Scale for Depression (HRSD) score of at least 14. Exclusion criteria included borderline and antisocial personality disorder, which were ruled out using the Structured Clinical Interview for DSM-IV for Axis II disorders (First et al., 1997). Participants were also excluded if they had herbal drug or medication use within the previous 8 weeks, with the exception of a SSRI, for which the exclusion period was 2 weeks prior to scanning. One subject was previously resistant to fluoxetine treatment, which was stopped 6 weeks prior to the baseline PET scan (standard practice for switching from fluoxetine to moclobemide treatment, when fluoxetine has been ineffective). There were four other participants who stopped their previous antidepressant treatment between 2 and 4 weeks prior to the baseline [^11^C]harmine PET scan, and received the following treatments as total daily doses (n = 1 each): Cymbalta (120mg), desvenlafaxine (50mg), Zoloft (50mg), and Effexor (37.5mg). It has been previously demonstrated that the technique used in the present work is not affected by SSRIs, as would be expected given their pharmacological specificity (Meyer et al., 2009). The therapeutic effect of SSRIs may therefore reflect countering downstream consequences of greater serotonin metabolism (Meyer et al 2009). Additional exclusion criteria included current or past substance abuse, nicotine abuse (cigarette smoking) within the past year (since recent cigarette smoking may bias MAO-A V_T_; Fowler et al., 1996; Bacher et al., 2011), positive urine toxicology drug screen, current perimenopause, current postmenopause, or positive urine pregnancy test (women). All participants underwent a urine toxicology drug screen on the day of eligibility assessment and on the PET scan days. To avoid conditions which could potentially bias MAO-A V_T_, participants were required not to consume any caffeine on the day of the PET scan and were asked not to use any over-the-counter medications or consume any alcohol for 48 hours prior to the PET scan. Participants were also screened to rule out common medical conditions associated with MDD through plasma sampling for protein, calcium, and thyroid stimulating hormone. No occupancy data from the participants of this study has been previously published. After complete description of the study to the subjects, written informed consent was obtained. The study was approved by the Research Ethics Board for Human Subjects at the Centre for Addiction and Mental Health, University of Toronto, in accordance with the Declaration of Helsinki.

### Imaging

For each PET scan, 370 MBq of [^11^C]harmine was administered as a bolus intravenously. An automatic blood sampling system was used to measure arterial blood radioactivity over the first 22 minutes of the scan. Manual samples were obtained at 2.5, 7.5, 15, 20, 30, 45, 60, and 90 minutes post injection. The method of generating arterial input function has been previously described (Ginovart et al., 2006). PET images were acquired using an high-resolution research tomograph PET camera (in-plane resolution; full width half maximum, 3.1mm; 207 axial sections of 1.2mm; Siemens Molecular Imaging) as previously described (Meyer et al., 2009). The frames consisted of 15 frames of 1 minute, followed by 15 frames of 5 minutes. [^11^C]Harmine doses were of high specific activity (scan 1 mean: 3156.59 mCi/μmol, standard deviation: 1983.87 mCi/μmol; scan 2 mean: 2720.34 mCi/μmol, standard deviation: 1251.44 mCi/μmol) and high radiochemical purity (scan 1 mean: 98.8%, standard deviation: 0.98%; scan 2 mean: 99.4%, standard deviation: 0.66%).

The primary regions of interest (ROIs) were the prefrontal cortex and anterior cingulate cortex, but additional regions with high MAO-A density and/or functional relevance to mood disorder symptoms were also included: the hippocampus, ventral striatum, dorsal putamen, thalamus, and midbrain (Saura et al., 1992, 1996; Ressler and Mayberg, 2007; Price and Drevets, 2010). For anatomical reference, each participant also underwent magnetic resonance imaging (GE Signa 1.5-T scanner; fast spoiled gradient echo, T_1_-weighted image; x, y, z voxel dimensions, 0.78, 0.78, and 1.5mm, GE Medical Systems). The ROIs were primarily defined using the Duvernoy (1999) atlas, with the exception of the divisions of the dorsal putamen and ventral striatum, which are described by Mawlawi et al. (2001). Details describing the delineation of each ROI have been previously described in the supplemental section of Matthews et al. (2014). The ROIs were delineated on the MRIs using a semi-automated method based on a template and nonlinear transformations (Ashburner and Friston, 1997, 1999; Rusjan et al., 2006). This was followed by a refinement process based upon the gray matter probability (Ashburner and Friston, 1997; Rusjan et al., 2006). The MRI was co-registered to the summated PET image using a normalized mutual information algorithm, and the resulting transformation was applied to the ROIs. The ROIs were verified by visual inspection of the individual co-registered MRI and summated PET image.

MAO-A V_T_ represents the ratio at equilibrium of the concentration of radioactivity in tissue to that of plasma. It is an excellent predictor of MAO-A density, because approximately 85% of the volume of distribution is specifically bound to MAO-A (V_s_). MAO-A V_T_ may be validly and reliably measured with either an unconstrained two-tissue compartment model or with the Logan method with arterial sampling (Ginovart et al., 2006), and the latter was applied in this study. Noise-induced bias of the Logan method has been shown to be negligible at the noise level of [^11^C]harmine regional time activity curves (Ginovart et al., 2006; Meyer et al., 2009).

Overall brain drug occupancy and the distribution volume of the non-displaceable compartment (V_nd_) were estimated from the slope and intercept of the Lassen Plot (Lassen et al., 1995; Cunningham et al., 2010). For this, V_T_ at baseline is plotted as a value on the x-axis and V_T_ (at baseline) - V_T_ (during treatment) is plotted as a value on the y-axis, which gives an equation in the form of y = mx - b. The Lassen plot assumes that V_nd_ is constant across brain regions and does not change by the treatment between PET scans, assumptions consistent with the two-tissue compartment kinetic analysis (Ginovart et al., 2006). Using the Lassen plot estimation for V_nd_, V_S_ was estimated as V_T_ - V_nd_ and occupancy was then calculated for each individual brain region [Occupancy = (V_S_(at baseline) – V_S_(during treatment))/V_S_(at baseline)]. An example of a typical Lassen Plot can be seen in Supplementary Figure S1.

### Statistical Analysis

To determine the dose-occupancy relationship for moclobemide, occupancy data were fit using the hyperbolic equation F(x) = a(x/[b+x]) with a (0,0) point included for each individual brain region (Sigmaplot v. 11, Systat). Also, a multivariate analysis of co-variance (MANCOVA) was applied with total daily dose as the covariate and the brain regions of interest (including the prefrontal cortex, anterior cingulate cortex, ventral striatum, dorsal putamen, thalamus, midbrain, and hippocampus) as the dependent variables (IBM SPSS Statistics v. 20).

A multivariate analysis of variance (MANOVA) was used to compare the regional occupancy across three groups: low-dose moclobemide (300 to 600mg total daily dose), higher-dose moclobemide (900 to 1200mg total daily dose), and phenelzine (45 to 60mg total daily dose). Mean occupancy for higher doses of moclobemide (900–1200mg total daily dose), average clinical doses of phenelzine (45–60mg total daily dose), and average doses of moclobemide (300–600mg total daily dose) were calculated and reported with a 95% confidence interval (CI) of the difference (IBM SPSS Statistics v. 20).

## Results

### Demographics

The three groups (total daily moclobemide dose of 300mg to 600mg, total daily moclobemide dose of 900mg to 1200mg, total daily phenelzine dose of 45 mg to 60mg) had similar depression severity scores on the 17-item HDRS prior to treatment [F_(2,24)_ = 0.04, *p* = 1.0], similar rates of reversed neurovegetative symptoms [X^2^_(2)_ = 0.05, *p* = 1.0], and were of similar ages [F_(2,24)_ = 0.9, *p* = 0.4]. As would be expected given these similarities (Chiuccariello et al., 2014), they also had similar regional baseline MAO-A V_T_ values across the ROIs, including the prefrontal cortex, anterior cingulate cortex, ventral striatum, dorsal putamen, thalamus, midbrain, and hippocampus [F_(14,32)_ = 1.079, *p* = 0.41]. However, the number of previous treatments [F_(2,24)_ = 3.1, *p* = 0.06] and number of classes of previous antidepressant trials differed [F_(2,24)_ = 3.6, *p* = 0.04], consistent with the assignment criteria (see Table 1).

### Dose-Occupancy Relationship of Moclobemide

**Figure 1.**
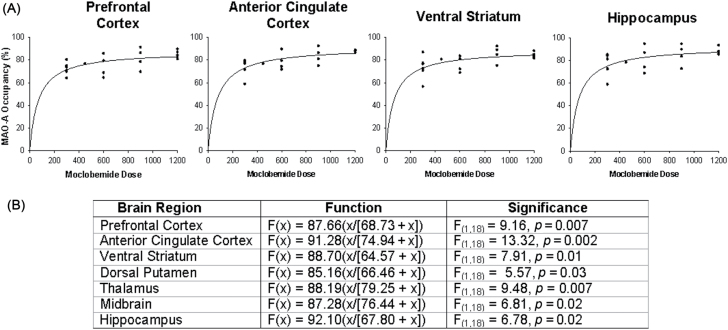
Relationship between monoamine oxidase-A occupancy and dose of moclobemide. The data were fit using the hyperbolic equation F_(0,0)_ = a(x/[b + x]). (A) Depicted here are a selected number of brain regions; however, the model significantly fit the data in each brain region tested. (B) Relationship between monoamine oxidase-A occupancy and dose of moclobemide for each brain region fit using the hyperbolic equation F_(0,0)_ = a(x/[b + x]).

**Figure 2.**
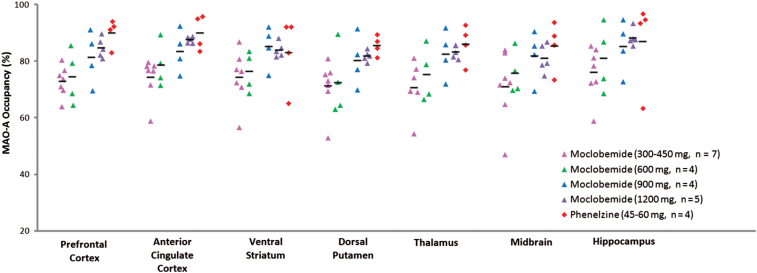
Monoamine oxidase-A occupancy higher in high doses of moclobemide (900–1200mg) and phenelzine (45–60mg) than low doses of moclobemide (300–600mg). There was a significant main effect of doses of moclobemide (1200mg and 900mg) and phenelzine (45 and 60mg) on occupancy across brain regions sampled when compared to the average clinical doses of moclobemide [300, 450, and 600mg; MANOVA, F_(7,16)_ = 3.94, *p* = 0.01].

The dose-occupancy relationship of moclobemide fit a hyperbolic function F(x) = a(x/[b + x]) were highly significant [F_(1,18)_ = 5.57 to 13.32, *p* = 0.002 to 0.03]. Values for a ranged from 85–92% (all regions, *p* < 0.0001) and b ranged from 64–79mg (range from *p* < 0.01 to *p* = 0.05; Figures 1 and 2). The mean occupancy ranged from 71.48±9.12% at a total daily dose of 300mg to 85.40±1.91% at a total daily dose of 1200mg. Consistent with this, applying dose as the covariate and the prefrontal cortex (PFC), anterior cingulate cortex (ACC), ventral striatum (VS), dorsal putamen (DP), thalamus, midbrain, and hippocampus occupancy as the dependent variables, a MANCOVA revealed a significant main effect of moclobemide dose on MAO-A occupancy [F_(7,12)_ = 5.58, *p* = 0.006].

### MAO-A Occupancy of Three Groupings (Total Daily Dose of 300 mg to 600 mg Moclobemide, Total Daily Dose of 900 mg to 1200 mg Moclobemide, Total Daily Phenelzine Dose of 45 mg to 60 mg)

A MANOVA revealed a significant main effect of higher doses of moclobemide (900mg and 1200mg) and phenelzine (45 and 60mg) on occupancy across brain regions sampled when compared to the average clinical doses of moclobemide [300–600mg; MANOVA main effect: F_(7,16)_ = 3.94, *p* = 0.01, regional comparisons: t = 2.27–4.28, *p* ≤ 0.001–0.03]. The mean brain MAO-A occupancy by moclobemide at average daily dose (300–600mg total daily dose, n = 11) was 74.23±8.32% (CI: 68.64–79.82%). The mean MAO-A occupancy by moclobemide at higher doses (900–1200mg total daily dose, n = 9) was 83.75±5.52% (CI: 79.50–88.0). The mean MAO-A occupancy by phenelzine (45–60mg total daily dose, n = 4) was 86.82±6.89% (CI: 75.86–97.78; Figure 2). The participant taking a total daily dose of 22.5 mg phenelzine had a MAO-A occupancy of 35.26% and was excluded from analyses, as this dose is below the minimum therapeutic dose supported by clinical trials (McGrath et al., 1987; Birkenhager et al., 2004). The average MAO-A occupancy of typical treating doses of phenelzine (45–60mg total daily dose) with standard error for each region of interest was as follows: PFC, 90.13±2.44%; ACC, 90.16±3.10%; VS, 83.11±6.39%; DP, 85.61±1.73%; thalamus, 86.18±3.40%; midbrain, 85.48±4.32%; and hippocampus, 87.06±7.93%. There was a trend to a relationship between the plasma level of moclobemide and overall brain MAO-A occupancy across the entire range of doses [F_(1,19) =_ 4.117, *p* = 0.058]. In the 300–900mg groups, numerical dose [F_(1,15) =_ 9.050, *p* = 0.011] and time since last dose [F_(1,15) =_ 8.808, *p* = 0.012] significantly predicted plasma concentration of moclobemide. Plasma level did not predict overall brain occupancy across the phenelzine doses [45–60mg, F_(1,2)_ = 4.081, *p* = 0.181].

### Post Hoc Analysis of Relationship between MAO-A Occupancy in the Prefrontal and Anterior Cingulate Cortex and Remission

Post hoc univariate analysis demonstrated that occupancy in the PFC and ACC was predictive of remission (less than 7 on the 17-item HRSD), with the presence of post-treatment remission as a dependent variable [PFC, F_(1,24)_ = 6.21, *p* = 0.02; ACC: F_(1,24)_ = 7.08, *p* = 0.01]. As MAO-A occupancy in regions of interest are highly correlated, there was a similar relationship between occupancy and post-treatment remission for all other regions tested [F_(1,24)_ = 6.69–7.30, *p* = 0.01–0.02].

### Comparison of MAO-A V_T_ from the Logan Model and Two-tissue Compartment Model

As an additional analysis, a subset of data from the present study was evaluated with both the Logan model and the two-tissue compartment model (n = 10 subjects baseline condition, n = 10 subjects occupied condition, prefrontal cortex assessed). The correlations between methods was high (r ~ 0.97–0.99), the values were equivalent in the baseline condition, and there was a 2% underestimate of MAO-A V_T_ with the Logan method in the occupied condition.

## Discussion

The main findings are that MAO-A occupancy increased with a greater moclobemide dose and that MAO-A occupancy of two different antidepressant regimens applied in treatment-resistant MDE (higher doses of moclobemide [900–1200mg total daily dose] or phenelzine [45–60mg total daily dose]) were significantly greater than MAO-A occupancy of a regimen applied in MDE with minimal histories of treatment-resistance (lower dose moclobemide [300–600mg total daily dose]). These results have implications for choosing optimal MAO-A occupancy for novel antidepressants, the use of moclobemide and phenelzine treatment, and algorithm design of MAOIs in MDD.

The dosing of moclobemide typically applied in MDE with minimal histories of treatment resistance was associated with a MAO-A occupancy of 74% and the dose of moclobemide or phenelzine applied in MDE with histories of treatment resistance was associated with a MAO-A occupancy of 84%, suggesting that when designing new MAO-A inhibitors, reaching a 74% occupancy is a suitable target for a novel first-line MDE antidepressant but reaching the 84% occupancy is a suitable target for MDEs with histories of treatment resistance. Given that the doses of moclobemide at 900mg and 1200mg are associated with a requirement of mild dietary tyramine restriction and the other clinically-available MAOIs (phenelzine and tranylcypromine) at any dose require a stringent dietary tyramine restriction, there is presently a therapeutic gap such that there is no MAOI that achieves an 85% occupancy without requiring some level of tyramine restriction (Simpson and Gratz, 1992; Dingemanse et al., 1998; Magder et al., 2000; Berlin et al., 1989). While it might seem contradictory to recommend a higher MAO-A occupancy for treatment since MAO-A density in MDE is elevated 35 to 40%, these numbers need not match for at least a couple of reasons. First, the elevation in MAO-A level may have been present for months to years with a number of downstream effects, and an antidepressant clinical trial is only 6 weeks. Second, there are a number of therapeutic targets in MDE, some of which may not be influenced solely by lowering available MAO-A, so raising monoamine levels excessively to reach other targets such as key signal transduction molecules may be important (Dwivedi, 2009).

The current findings also have implications for treatment algorithm design with MAOIs. With regards to prescribing moclobemide, the data demonstrates that the typical dose-occupancy curve has not reached a plateau across the doses tested, so after non-response at a lower dose there is a logical reason to expect greater target engagement at a higher dose. With regards to switching from higher-dose moclobemide to phenelzine, since MAO-A occupancies are reasonably similar, the best rationale would be to obtain additional targets with phenelzine, such as inhibition of MAO-B or primary amine oxidase or elevation of GABA levels (Baker et al., 1991, 1992; Holt et al., 2004). Another clinically relevant point is that low doses of irreversible MAOIs are unlikely to achieve 100% occupancy, or even a substantial occupancy, since the phenelzine occupancy for doses between 22.5mg and 60mg daily ranged from 35% to 88%. This is an important issue since there is a widespread assumption that irreversible MAOI treatments obtain high occupancy at low dose, an assumption inherent in the selection of low-dose tranylcypromine for the STAR*D trial (McGrath et al., 2006; Nolen et al., 2007).

We found that plasma levels of moclobemide did not predict brain occupancy of MAO-A, which might be explained by at least two broad reasons. First, it is known that the direct binding of moclobemide to MAO-A in tissue homogenates *in vitro* does not fully explain its effects on MAO-A function *in vivo*. Moclobemide is a mixed competitive/non-competitive inhibitor of MAO-A, yet, despite the short elimination half-life in plasma (1–2h) and rapid recovery of MAO-A enzymatic function *in vitro* (recovery after 4 hours), the effect of inhibiting MAO-A activity lasts for 8–16 hours *in vivo* (Da Prada et al., 1989) via a process not yet identified. Second, it is possible that the levels of drug in plasma are not reflecting the levels of drug in the central nervous system. For example, MAO-A is primarily metabolized by the CYP2C19 enzyme, which is also present in the brain and the liver (McFayden et al., 1998; Ingelman-Sundberg et al., 2014). If the concentration of moclobemide in the brain is substantially influenced to a variable extent across different subjects via brain CYP2C19 enzymes, this might account for a discrepancy between peripheral plasma drug level and brain MAO-A occupancy.

It is interesting that none of the dosing regimens reached an occupancy of 100% and the occupancy hyperbolic curve fits suggest that 100% occupancy would not be achievable at even very high doses of moclobemide. This is consistent with *in vivo* occupancies for some therapeutics for other targets: for example, maximal D_2_ occupancies have been observed for clozapine at 60% (Nordstrom et al., 1995) and 90% at the serotonin transporter for most SSRIs (Meyer et al., 2004; Voineskos et al., 2007). There are a number of reasons as to why these medications may not reach 100% occupancy in the brain, which may include presence of a subpopulation of MAO-A proteins that are accessible to [^11^C]harmine but not moclobemide or phenelzine, a dramatically increased synthesis of MAO-A under high-occupancy states, and/or, in the case of moclobemide, reduced intracellular removal of MAO-A protein during high-occupancy states. One theoretical reason for the maximum predicted occupancy being less than 100% for moclobemide could be that the MAO-A V_T_ values are reduced by clinical response, providing an additive effect to the occupancy measurement to a greater extent at lower doses, thereby leading to a reduction in the maximum predicted occupancy. This is unlikely due to the nature of the relationship between MDE and MAO-A V_T_: events associated with the onset of a MDE, such as elevated glucocorticoids from chronic stress or reductions in estrogen in early postpartum or in perimenopause are associated with elevated MAO-A V_T_, levels and/or activity (Meyer et al., 2006; Ou et al., 2006b; Sacher et al., 2010; Rekkas et al., 2014), and greater severity of MDE is associated with higher MAO-A V_T_ in brain grey matter regions, particularly the PFC and ACC (Chiuccariello et al., 2014). Elevated MAO-A V_T_ and/or density persists into remission, although the highest levels of MAO-A V_T_, particularly in the PFC and ACC, are associated with a recurrence of MDEs (Meyer et al., 2009; Johnson et al., 2011). This elevation in MAO-A V_T_ is presently viewed as a scar-like effect and is unchanged, even when measured before and after acute SSRI treatment (Meyer et al., 2009; Johnson et al., 2011). Thus, the level of clinical response to moclobemide would not be expected to affect MAO-A V_T_, even though the converse is implicitly true given the efficacy of moclobemide. Another theoretical reason to consider for the maximum predicted occupancy of moclobemide being less than 100% is that the Logan method underestimated the MAO-A V_T_ values more in the occupied condition. This is unlikely, because the underestimate from the Logan method on MAO-A V_T_ derived from region-based time activity curves is typically negligible. In the present study, the results of the Logan and two-tissue compartment model were assessed in a subset of 10 subjects with both methods: there was no difference in MAO-A V_T_ at baseline and there was a 2% underestimate of MAO-A V_T_ with the Logan method in the occupied condition. Hence, it is more likely that the maximum occupancy of 100% is difficult to reach with moclobemide.

A potential limitation is that this study was not designed to examine the relationship between occupancy and therapeutic response. While this is theoretically possible, most clinical trials differentiating antidepressants from placebo investigation require 100 or more subjects in each treatment group (Thase, 1999; Gibertini et al., 2012), which is not generally feasible for PET imaging studies due to the cost. However, it is interesting that in a post hoc analysis, occupancy was predictive of remission as measured by the HRSD. Furthermore, it should be noted that four of five individuals that achieved remission had an occupancy above 82%, whereas only one had an occupancy below 82%. Another potential limitation of this study is that we did not focus on subclinical doses of moclobemide or phenelzine, which would have provided more information about the saturation curve.

In conclusion, this is the first study to evaluate the relationship between dose and MAO-A occupancy for moclobemide and to investigate the occupancy of typical clinical doses of phenelzine. Our findings have direct implications for MAO-A inhibitor development. To design a MAO-A inhibitor as a first line for MDE, our results suggest that an occupancy of at least 74% is required, since this corresponds to the occupancy of low doses of moclobemide, whereas to design a MAO-A inhibitor for more treatment-resistant MDE an occupancy of at least 84% is desirable because this corresponds to the occupancy of moclobemide and phenelzine dosing used in these clinical situations. Our data also demonstrates that a plateau in the dose occupancy relationship for moclobemide has not yet been reached across typical treating doses; hence, for attempting to engage more targets with treatment, there is a logical rationale for raising the dose of moclobemide from a 300 to 600mg daily dose to a 900 to 1200mg total daily dose. Also, since MAO-A occupancy with phenelzine treatment of 45 to 60mg daily is comparable to moclobemide at 900 to 1200mg daily, the main rationale for switching across these treatments is to engage additional targets with phenelzine rather than greater occupancy of MAO-A. Future studies of new MAO-A inhibitors should incorporate MAO-A occupancy measurement to assess whether they offer distinct advantages of target engagement relative to their side effects as compared to presently available treatments.

## Supplementary Material

For supplementary material accompanying this paper, visit http://www.ijnp.oxfordjournals.org/

## Statement of Interest

This work was supported by grants from the Canadian Institutes of Health Research (CIHR) and salary support from the Ontario Mental Health Foundation and CIHR. Drs Meyer, Wilson, and Houle have received operating grant funding for other studies from Eli Lilly, GlaxoSmithKline, Bristol-Myers Squibb, Lundbeck, and SK Life Science in the past 5 years. Dr Meyer has consulted with several of these companies as well as Sepracor, Trius Therapeutics, and Mylan Inc. None of these companies participated in the funding, design, or execution of this study or writing the manuscript. Dr Meyer is developing natural health products to treat high MAO-A states. Dr Meyer is applying for patents to implement measures of utilizing MAO to diagnose or treat mood disorders. It is likely that companies which make medications affecting monoamine receptor or monoamine oxidase binding will seek collaborations with these investigators in the future. Dr Baker has had research funding from Pfizer and Sinoveda Canada in the past 5 years and has collaborated with Phenomenome Discoveries, but none of that research is related to the present project. Dr Kish receives research funding from US NIH NIDA Grant Number DA 25096. All other authors declare no competing interests.

## Supplementary Material

Supplementary Figure S1
