# Supplementary material for: Monoamine Oxidase-A Occupancy by Moclobemide and Phenelzine: Implications for the Development of Monoamine Oxidase Inhibitors
Source: Int J Neuropsychopharmacol. 2015 Aug 27;19(1):pyv078. doi: 10.1093/ijnp/pyv078 (PMC4772270; doi:10.1093/ijnp/pyv078)
Supplement: Supplementary Figure S1 [file MAOAOccupancy_Supplemental_Section_LChiuccariello.doc]

**Monoamine Oxidase-A Occupancy by Moclobemide and Phenelzine:**

**Implications for the Development of Monoamine Oxidase Inhibitors**

##### *Short title: MAO-A Occupancy by Moclobemide and Phenelzine*

***SUPPLEMENTAL SECTION***

**METHODS**

**Moclobemide and Phenelzine Plasma Assays**

*Moclobemide:* Buproprion was added as an internal standard to all tubes. Calibration curves, run in parallel with each assay, were prepared using tubes each containing a different, known amount of authentic moclobemide and a known, fixed amount of bupropion. The same fixed amount of bupropion was added to each plasma sample and the ratios of moclobemide to bupropion in the extracted plasma samples were compared to those on the calibration curve to determine the amount of moclobemide in each plasma sample. Standards, blanks and samples were basified to pH 11.00 with 25% K_2_CO_3_ and extracted with ethyl acetate. The organic layer was removed and taken to dryness under vacuum. The residue was reconstituted in toluene and injected on to an Agilent 7890B GC system coupled to a 7000C GC/MS triple quad in the electron impact (EI) mode. An HP5-MS UI (Agilent) column was employed in the assay. The oven temperature was held at 100 degrees C for 1 min and then ramped at 15 degrees C/min for a final temperature of 295 degrees C. Moclobemide and buproprion eluted at 15.15 and 8.6 minutes respectively. The transitions *m/z* 140>112 and 139>111, for moclobemide and buproprion respectively, were recorded in multiple reaction monitoring mode for identification and quantification. Agilent Mass Hunter software was used for all instrument control and data manipulation. The r^2^ value for each calibration curve was >0.99.

*Phenelzine:* Phenelzine was assayed by a modification of the method of Rao et al., (Rao et al., 1987) using benzylamine as the internal standard. A calibration curve, which was constructed in parallel with each assay, was prepared by using several tubes, each containing a different, known amount of authentic phenelzine and a fixed, known amount of benzylamine. The same fixed amount of benzylamine as used in the calibration curve was added to each plasma sample. The ratios of derivatized phenelzine to derivatized benzylamine in the samples were compared to the ratios in the calibration curve to determine the amount of phenelzine in each plasma sample. Briefly, samples, standards and blanks were basified, extracted and derivatized with pentafluorobenzoyl chloride. The organic layer was saved and taken to dryness. The residue was reconstituted in toluene and a portion injected on to a HP-5MS column (Agilent) installed in an Agilent 6890 series GC system coupled to 5973 N mass spectrometer in the negative chemical ionization (NCI) mode. Oven conditions were as follows: initial temperature 100ᵒC, oven ramp 15ᵒC/min, final temperature 295ᵒC (held for 10 min). The retention times of derivatized benzylamine (internal standard) and derivatized phenelzine were 9.1 and 12.48 minutes respectively. Single ion monitoring (benzylamine m/z 281; phenelzine m/z 504) was used for identification and quantification. MSD Chemstation was used for instrument control and data analysis. The r^2^ value for each calibration curve was >0.99.

**RESULTS**

**Figure S1. A Typical Lassen Plot used to Determine Non-Specific Binding.** The equation of the line allows for the calculation of V_ND_ from the x-intercept. Using the Lassen plot estimation for V_ND_, V_S_ was estimated as V_T_ – V_ND_ and occupancy was then calculated for each individual brain region (MAO-A Occupancy = (V_S_(at baseline) – V_S_(during treatment))/V_S_(at baseline)*100).

**DISCUSSION**

*Further discussion on effect of chronic MAOI treatment upon MAO-A occupancy measurement:*

An important point of interpretation is that [^11^C]harmine binds to the centre of the functional cavity of MAO-A (Son et al., 2008) and the occupancy measure reflects a change in the specific binding of [^11^C]harmine. Interesting aspects of this measure is its high relevance to true *in vivo* conditions and it also allows for comparisons of effect between irreversible and reversible MAOIs. However, it does reflect more than the immediate binding effects of antidepressant drug to MAO-A. For example, chronic effects of treatment on the overall density of MAO-A or on the affinity of MAO-A for [^11^C]harmine, may also influence the occupancy. Given the limitations of a one month period for healing after arterial sampling in humans, future studies in preclinical models might aid in determining whether chronic MAO inhibition has different effects on overall MAO-A density or affinity as compared to acute treatment.

**REFERENCES**

Rao TS, Baker GB, Coutts RT, Yeung JM, McIntosh GJ, Torok-Both GA (1987) Analysis of the antidepressant phenelzine in brain tissue and urine using electron-capture gas chromatography. J Pharmacol Methods 17:297-304.

Son SY, Ma J, Kondou Y, Yoshimura M, Yamashita E, Tsukihara T (2008) Structure of human monoamine oxidase A at 2.2-A resolution: the control of opening the entry for substrates/inhibitors. Proceedings of the National Academy of Sciences of the United States of America 105:5739-5744.
